# Nucleic Acids Delivery Into the Cells Using Pro-Apoptotic Protein Lactaptin

**DOI:** 10.3389/fphar.2019.01043

**Published:** 2019-09-18

**Authors:** Olga Chinak, Ekaterina Golubitskaya, Inna Pyshnaya, Grigory Stepanov, Evgenii Zhuravlev, Vladimir Richter, Olga Koval

**Affiliations:** ^1^Laboratory of Biotechnology, Institute of Chemical Biology and Fundamental Medicine, Siberian Branch of the Russian Academy of Sciences, Novosibirsk, Russia; ^2^Laboratory of Genome Editing, Institute of Chemical Biology and Fundamental Medicine, Siberian Branch of the Russian Academy of Sciences, Novosibirsk, Russia; ^3^Department of Natural Sciences, Novosibirsk State University, Novosibirsk, Russia; ^4^Laboratory of Biomedical Chemistry, Institute of Chemical Biology and Fundamental Medicine, Siberian Branch of the Russian Academy of Sciences, Novosibirsk, Russia

**Keywords:** nucleic acids, cell penetrating peptides, lactaptin, gene delivery, snoRNA

## Abstract

Cell penetrating peptides (CPP) are promising agents for transporting diverse cargo into the cells. The amino acid sequence and the mechanism of lactaptin entry into the cells allow it to be included into CPP group. Lactaptin, the fragment of human milk kappa-casein, and recombinant lactaptin (RL2) were initially discovered as molecules that induced apoptosis of cultured cancer cells and did not affect non-malignant cells. Here, we analyzed the recombinant lactaptin potency to form complexes with nucleic acids and to act as a gene delivery system. To study RL2-dependent delivery, three type of nucleic acid were used as a models: plasmid DNA (pDNA), siRNA, and non-coding RNA which allow to detect intracellular localization through their functional activity. We have demonstrated that RL2 formed positively charged noncovalent 110-nm-sized complexes with enhanced green fluorescent protein (EGFP)-expressing plasmid DNA. Ca^2+^ ions stabilized these complexes, whereas polyanion heparin displaced DNA from the complexes. The functional activity of delivered nucleic acids were assessed by fluorescent microscopy using A549 lung adenocarcinoma cells and A431 epidermoid carcinoma cells. We observed that RL2:pDNA complexes provided EGFP expression in the treated cells and that strongly confirmed the entering pDNA into the cells. The efficiency of cell transformation by these complexes increased when RL2:pDNA ratio increased. Pre-treatment of the cells with anti-RL2 antibodies partly inhibited the entry of pDNA into the cells. RL2-mediated delivery of siRNA against EGFP was analyzed when A549 cells were co-transfected with EGFP-pDNA and RL2:siRNA complexes. siRNA against EGFP efficiently inhibited the expression of EGFP being delivered as RL2:siRNA complexes. We have previously demonstrated that non-coding U25 small nucleolar RNA (snoRNA) can decrease cell viability. Cancer cell transfection with RL2-snoRNA U25 complexes lead to a substantial decrease of cell viability, confirming the efficiency of snoRNA U25 delivery. Collectively, these findings indicate that recombinant lactaptin is able to deliver noncovalently associated nucleic acids into cancer cells *in vitro*.

## Introduction

Successful delivery of exogenous DNA and RNA molecules into cells has major implications for gene-based technologies and therapeutic approaches. Since free DNA and RNA are not readily taken up by cells, various delivery systems were developed during the last twenty years (Bolhassani, 2011). Delivery molecules or vesicles help nucleic acids to cross biological barriers and prevent enzymatic degradation by endogenous nucleases. Besides the lipid-based delivery system, the polymer-based delivery system or inorganic nanoparticles, peptide carriers can be also used for nucleic acids transport into the cells (Zorko and Langel, 2005; Rathnayake et al., 2017). Among peptide carriers, the most promising are short amphipathic and cationic peptides, which translocate themselves across membranes and are collectively named as cell-penetrating peptides (CPP) (Hoyer and Neundorf, 2012; Morris and Labhasetwar, 2015). There is no unique classification for CPPs yet, and usually, they are classified according to their origin, specificity to cargo, and amino acid composition. In 1997, Morris et al. demonstrated that 27-residue CPP peptide vector being premixed with single- or double-stranded deoxyoligonucleotides efficiently delivered them into cultured mammalian cells (Morris et al., 1997). Later, two CPPs—transportan and penetratin—successfully delivered 21-mer peptide nucleic acid (PNA) complementary to the human galanin receptor type 1 mRNA resulted in the suppression of galanin receptor (Pooga et al., 1998). Thereby, CPPs deliver functionally active nucleic acids into the cells.

Lactaptin, an 8.6-kDa proteolytic fragment of human milk kappa-casein, selectively kills various tumor cells while it is used in micromole concentration (Semenov et al., 2010; Koval et al., 2012). We have earlier demonstrated that recombinant lactaptin (RL2) penetrates into cells partly through lipid raft-mediated dynamin-independent pinocytosis and partly through direct penetration across the plasma membrane (Chinak et al., 2016). The comparison of RL2 primary structure and the mechanism of its penetration into the cell suggests that it can be assigned to the CPP.

In the present work, we analyzed if lactaptin analog forms complexes with nucleic acids and if it acts as a delivery system with no cytotoxic effects.

## Results

### RL2 Forms Non-Covalent Complexes With Plasmid DNA

CPPs, like other cationic proteins or peptides, can condense DNA through their cationic residues by non-covalent electrostatic interactions (Tecle et al., 2003). Since RL2 has the properties of cell-penetrating peptides, its ability to deliver cargo molecules into the cells was studied using a plasmid DNA (pEGFP). This plasmid contains a coding sequence of the enhanced green fluorescent protein (EGFP) under the control of the cytomegalovirus (CMV) immediate early promoter, allowing expression of EGFP in eukaryotic cells.

The charge ratio N/P or peptide nitrogen per nucleic acid phosphate of RL2:pEGFP complexes was calculated as described in the Materials and Methods section for the analysis of the efficient ratio of RL2 and pDNA. Here, to optimize empirically the ratio of RL2 and pEGFP for the non-covalent complexes (RL2:pEGFP) formation, we have varied the RL2 amount, whereas pDNA amount was constant within N/P ratio of 0.5 to 5. UV-vis spectroscopy analysis of complexes (**Supplementary Figure 1**) demonstrates that RL2:pDNA complexes have maximum absorbance in the range of 260 to 280 nm, whereas pDNA has an absorption maximum at 260 nm and RL2 at 280 nm.

**Figure 1:**
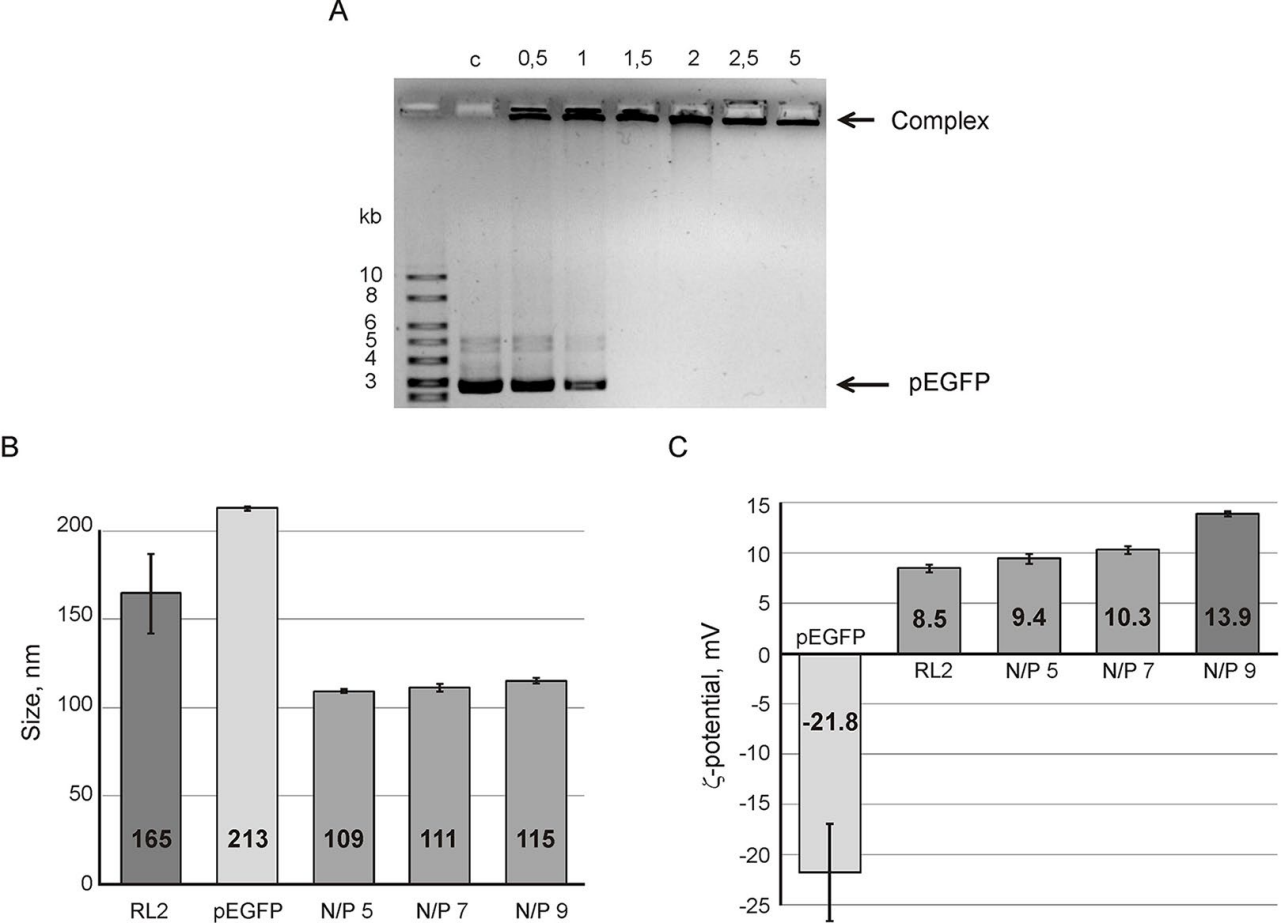
Analysis of RL2:pEGFP complex formation. **(A)** Gel retardation assays of the RL2:pEGFP complex. N/P ratios of RL2/pDNA charge have varied from 0.5 to 5 in reaction mixtures; C- control (free pDNA). Particle size **(B)** and ζ potential **(C)** of RL2:pEGFP complexes were analyzed for N/P ratios 5, 7, and 9. Reactions were performed in MES buffer for 5 min at 37°C. Particle size was estimated by the DLS technique. The data are representative of three independent repeats and are shown as the mean ± SD.

The efficiency of complex formation was analyzed by agarose gel retardation assay. Data obtained (**Figure 1A**) shows the conditions when pDNA movement retards in gel indicating complex formation. The increase of RL2 amount leads to the decrease of free plasmid DNA in a reaction mixture. Complete retardation was reached at a N/P charge ratio of five (**Figure 1A**). It indicates that all pDNA was totally involved into RL2:pEGFP complexes which had no mobility under the conditions of electrophoresis due to their larger size compared to the pores size, or the neutral charge.

To characterize RL2:pEGFP complexes, the hydrodynamic size and ζ-potential of the complexes were estimated using the dynamic light scattering (DLS) technique. **Figure 1B** shows that the complex size is near 110 nm with no significant differences for the charge ratio range from 5 to 9. The size of the RL2:pEGFP complex was smaller than the sizes of RL2 or pEGFP alone. Thus, when complexes form, both molecules condense.

Electrokinetic potential (ζ-potential) of pDNA and RL2 in a solution demonstrated a negative charge for DNA and a positive charge for RL2. For complexes, ζ-potential gradually increases with the N/P increasing from 5 to 9 (**Figure 1C**). We suppose that the increasing of ζ-potential is specified by the rising amount of surface-exposed cationic groups of RL2 molecules in RL2:pDNA complexes.

### Ca^2+^ and Heparin Change the Stability of RL2:pEGFP Complexes

**Figure 2:**
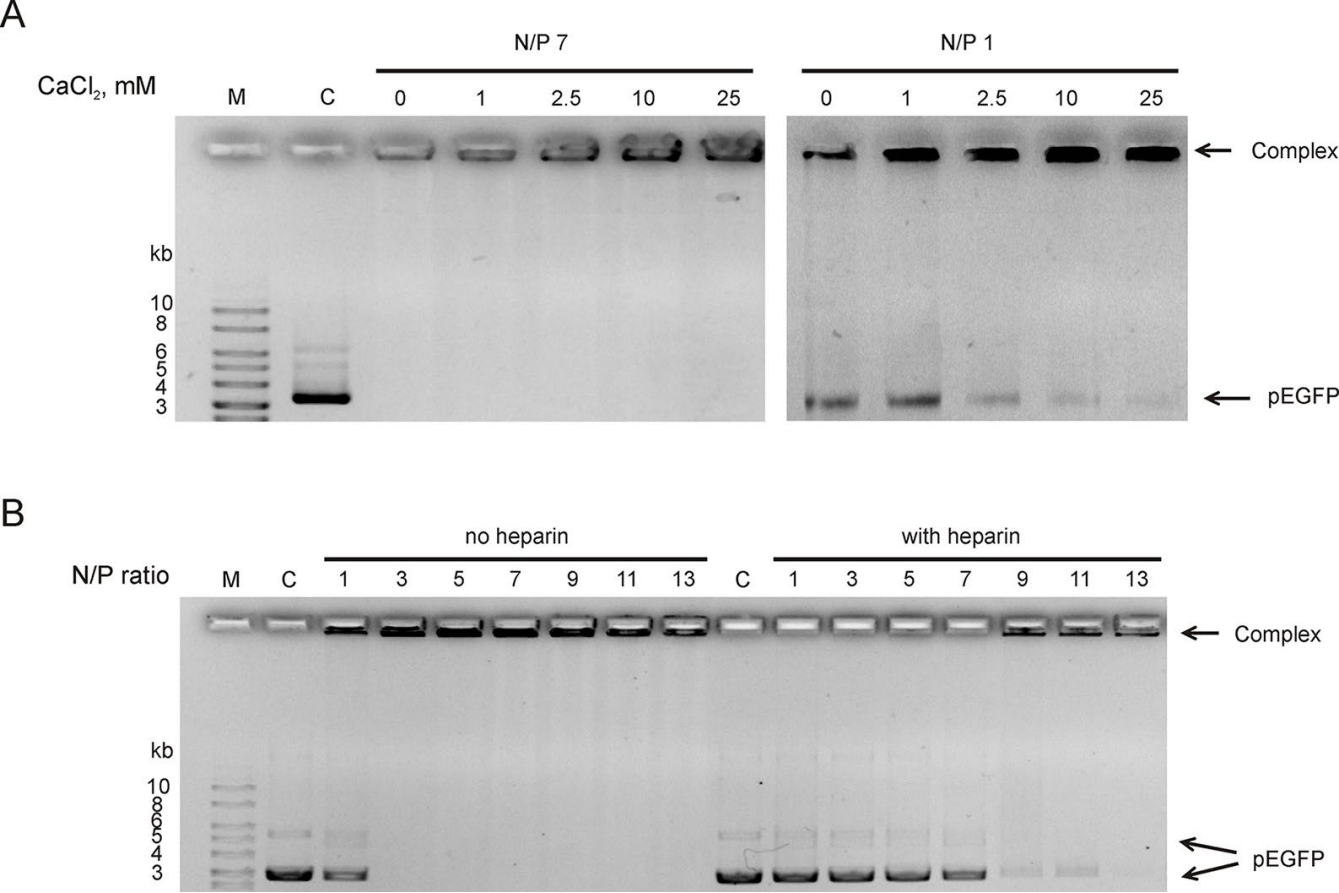
RL2:pEGFP complex analysis in the presence of Ca^2+^ **(A)** and heparin in ratio 2.4 Units/1 mkg pEGFP **(B)**. Gel retardation assays. C—free pDNA (150 ng); M—DNA ladder. Complexes were pre-formed and after that Ca^2+^ or heparin were added. Representative picture of three independent experiments.

Since RL2 originates from milk k-casein, it can be sensitive to Ca^2+^ ions concentration. It is known that environmental Ca^2+^ can stimulate aggregation or dissociation of casein’s micelles (Ye and Harte, 2013). To reveal the Ca^2+^ effects on the stability of RL2:pEGFP complex, complexes were formed under semi-extreme N/P ratios. Under N/P = 7, there was no free pDNA and Ca^2+^ did not release pDNA from the RL2:pEGFP complex (**Figure 2A**). Under N/P = 1, only half of pDNA molecules were in the RL2:pEGFP complex, and these conditions allow us to analyze Ca^2+^ concentration when total pDNA is incorporated in complex with RL2. The increase of Ca^2+^ concentration rises the amount of pDNA incorporated in the complex with RL2 (**Figure 2A**).

Heparin is a stronger polyanion than DNA; therefore, it can competitively bound to RL2, releasing plasmid from RL2:pEGFP complexes. Heparin sodium salt (2.4 units per 1 µg DNA) was added to pre-formed RL2:pEGFP complexes, and the appearance of free pDNA was analyzed. We showed that heparin releases pEGFP from the complexes with N/P ≤ 11 (**Figure 2B**). Complexes with high N/P ratio were more resistant to heparin.

Thus, Ca^2+^ divalent cations stabilize complexes and polyanion heparin destabilizes RL2:pEGFP complexes.

### Plasmid DNA Delivery

**Figure 3:**
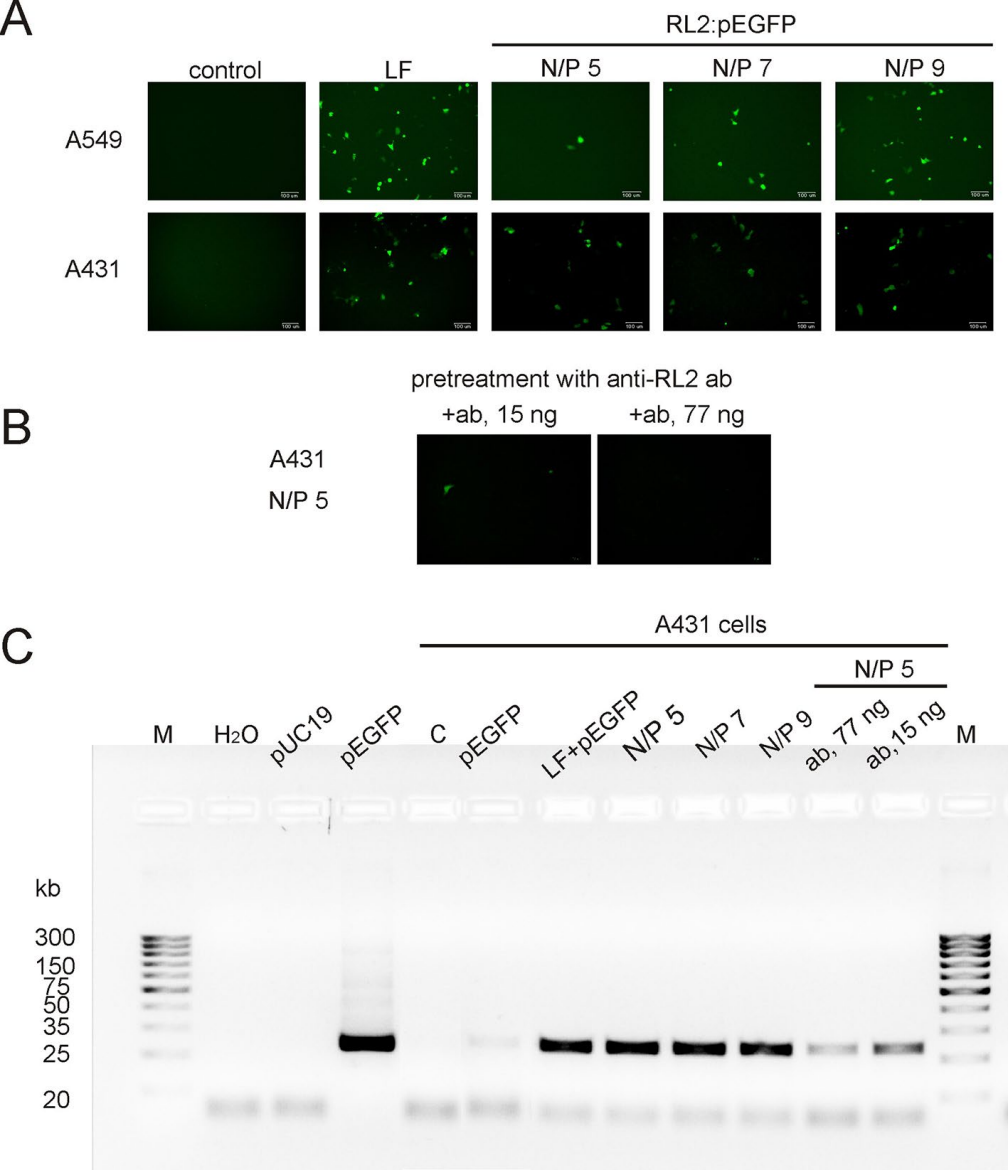
Transfection of cancer cells by RL2:pEGFP at charge ratios from 5 to 9. **(A)** Cells were incubated with LF—Lipofectamine 3000 and pEGFP, free pEGFP or RL2-pEGFP complexes with various N/P ratios and 24 h after EGFP was analyzed by fluorescent microscopy. **(B)** Cells were pre-treated with anti-RL2 antibodies (ab) for 3 h and next transected with a complex RL2:pEGFP. **(C)** Electrophoretic analysis of the products of RT-PCR reaction with EGFP-specific primers. Free pUC19 and pEGFP were used as matrixes in PCR reaction for the negative and positive controls, respectively. Representative pictures of three independent experiments.

To examine whether RL2 delivers pEGFP into human cancer cells the functional-based method was used. A549 and A431 cells were incubated with RL2:pEGFP complexes with various N/P ratios. After that, a reporter EGFP protein was analyzed by fluorescence microscopy. EGFP fluorescence was observed in the cells treated with complexes characterized by the high N/P ratio (**Figure 3A**). No fluorescence signal was seen when cells were treated with naked pEGFP, indicating that plasmid DNA itself did not enter into the cells under these conditions. The increase of RL2 content in complexes led to the increase of the number of cells with EGFP, and the highest EGFP fluorescence was in cells incubated with a RL2:pEGFP complexes with N/P = 9.

The entering of RL2:pEGFP complexes into the A431 cells was also evaluated by RT-PCR analysis with EGFP-specific primers using whole cellular RNAs as a matrixes. EGFP-specific PCR products were detected in cells treated with RL2:pEGFP complexes within N/P ratio of 5 to 9 (**Figure 3C**). No PCR product was seen when cells were transfected with pEGFP alone or with control pDNA.

Thus, RL2 provides translocation of plasmid DNA into the cells and saves pEGFP functional activity upon internalization. Pre-treatment of the cells with anti-RL2 antibodies partly inhibited the entry of pEGFP into the cells (**Figures 3B, C**).

### RL2:siRNA Complex Formation

**Figure 4:**
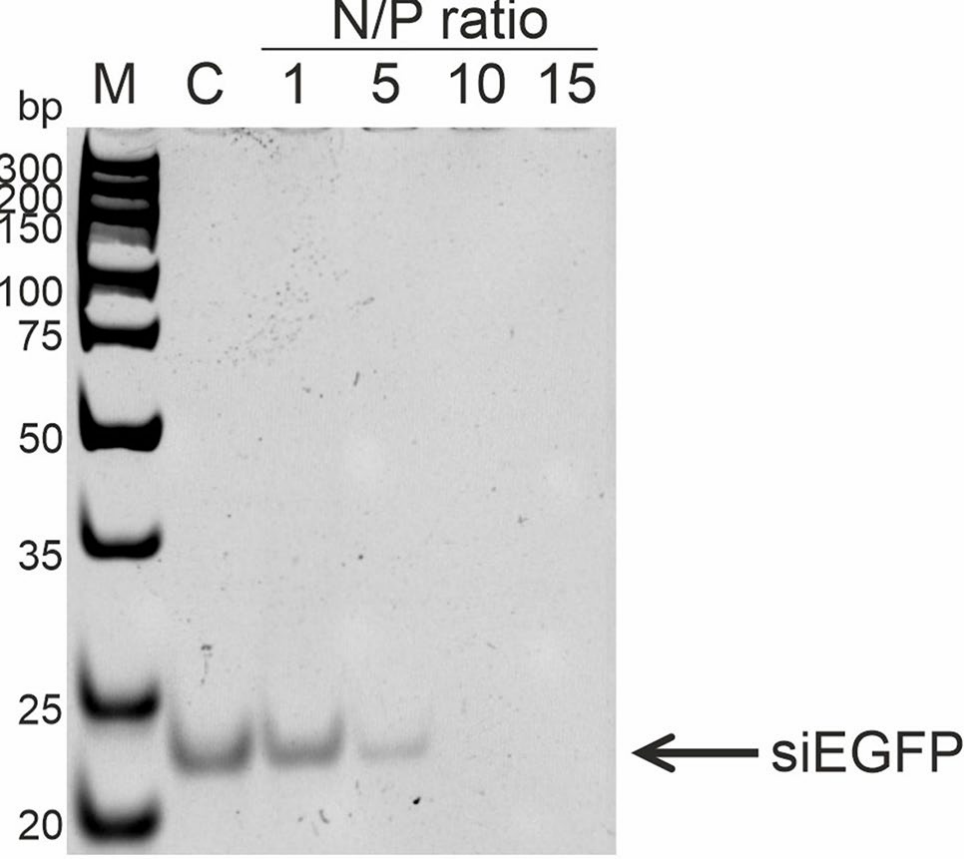
Analysis of RL2:siRNA complex formation. Gel retardation assays. c—free siRNA; M—DNA ladder. Representative picture of two independent experiments.

To analyze RL2:siRNA complex formation, the well-described anti-EGFP siRNA, which targets the EGFP gene coding region, was used (Nagy et al., 2003). The efficiency of complex formation was analyzed with N/P ratios of RL2:siRNA from 1 to 15. Gel retardation assay revealed that the free form of siRNA in the samples disappeared when the N/P ratio was 10 and higher (**Figure 4**). Thus, RL2 efficiently forms complexes with siRNA with N/P more than 5.

### siRNA Delivery

**Figure 5:**
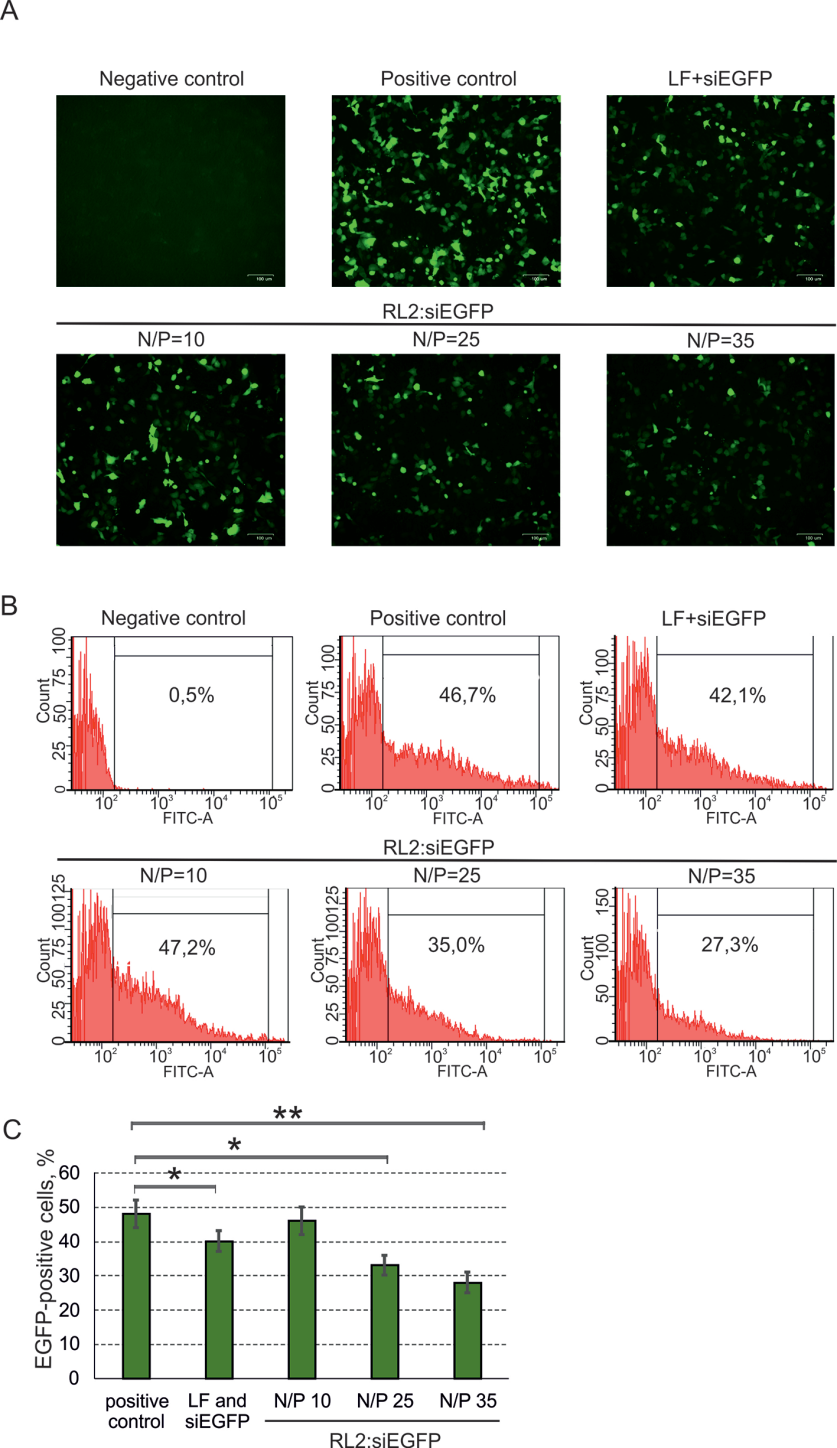
Specific activity of anti-EGFP siRNA delivered into A549 cells as a RL2:siRNA complex. LF—Lipofectamine 3000. Negative control—cells were treated with naked pEGFP; positive control—cells treated with pEGFP and LF. **(A)** Representative images of fluorescent microscopy data; **(B)** Representative images of flow cytometry analysis. Representative images of EGFP-positive gate excludes EGFP-negative cells (relative to negative control). **(C)** Quantitative evaluation of EGFP down-regulation. The data are representative of three independent repeats and are shown as the mean ± SD. *P < 0.05; **P < 0.03.

To study RL2-based siRNA delivery, at first, A549 cells were transfected with pEGFP, and then cells were incubated with pre-forming RL2:siRNA complexes. For the stage of pEGFP transfection, the Lipofectamine 3000 was used to avoid excessive RL2 amount for the cell treatment (positive control). Cells transected with naked pEGFP (negative control) demonstrate no EGFP fluorescent signal (**Figure 5A**). EGFP was down-regulated in the RL2:siRNA-treated cells when N/P was 25 or higher. Flow cytometry analysis of transfected cells confirmed the data obtained and showed approximately a 50% decrease of EGFP for the complex with N/P = 35 (**Figures 5B, C**). These experiments demonstrate the specific effects of anti-EGFP siRNA, allowing us to conclude that siRNA efficiently penetrates into the cells treated with RL2:siRNA complexes.

### Cytotoxic Activity of RL2:U25 snoRNA Complex

**Figure 6:**
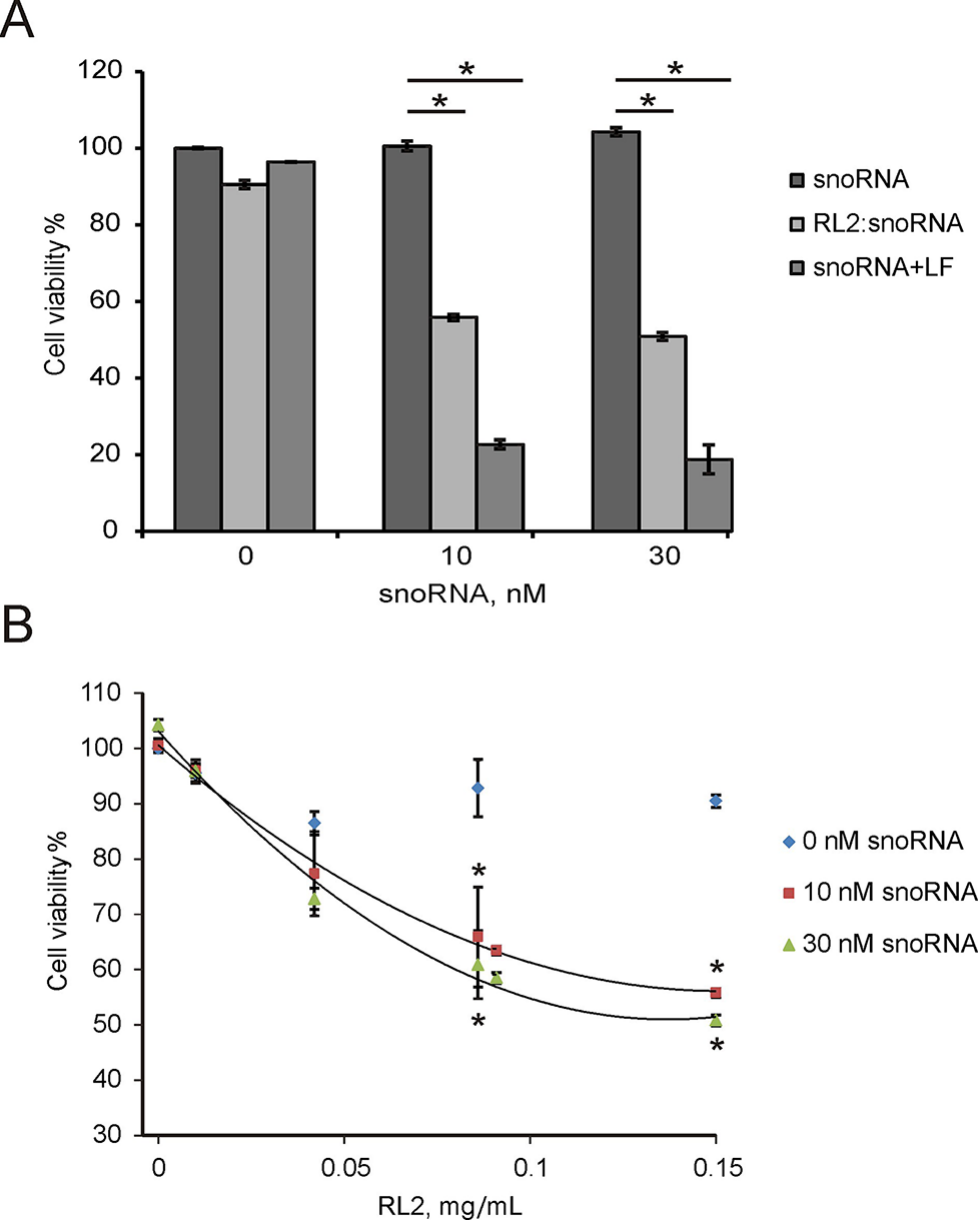
Cytotoxic activity of non-coding artificial analogue of U25 box C/D snoRNA in A549 cells. Cells were treated with RL2:snoRNA complexes, and after 48 h of incubation, cells viability was analyzed by MTT. LF—Lipofectamine 3000. **(A)** Comparison with LF-dependent delivery. *P < 0.05 compared with control (free RNA); **(B)** Concentration-response curves for the different RL2 concentration. The data are representative of three independent repeats. *P < 0.05 compared with control (only RL2 treatment).

Non-coding RNAs have been shown to modulate various cellular responses as well as induce death of cancer cells *in vitro* (Van et al., 2012; Stepanov et al., 2016). Earlier, we demonstrated that non-coding artificial analogue of U25 box C/D snoRNA (snoRNA U25) decreased the viability of various cancer cells *in vitro* (Nushtaeva et al., 2018). The cytotoxicity U25 snoRNA was in part due to the activation of inflammation-involved genes. U25 snoRNA is a single-stranded molecule in comparison with double-stranded pDNA or siRNA. The complex of RL2 with snoRNA was performed as described in the Materials and Methods section. For the experiments, RL2 was used in low-cytotoxic concentration. A549 cells were treated with RL2:snoRNA complex, and 48 h after MTT analysis revealed the decrease of cell viability (**Figure 6A**). **Figure 6A** demonstrates that Lipofectamine-delivered U25 snoRNA decreased the viability of treated cells more substantially than RL2-delivered. Concentration-response curves show the rise of cytotoxic effects with the rising of RL2 concentration only for RL2:snoRNA complexes, but not for free RL2 (**Figure 6B**). The increase of RL2 quantity in the complex elevates the penetration potency of such complexes, this seems to be the same as the other investigated complex of nucleic acids with RL2. Analyzing the data obtained, we conclude that the decrease of viability of treated cells was induced by snoRNA activity, and this efficiency correlated with the penetration potency of RL2:snoRNA complex.

## Discussion

Negative charge of DNA and RNA molecules hampers their movement across cellular membranes. To reach intracellular space, DNA or RNA molecules usually need to be packaged in vesicles or carriers. Usually, peptides are used as cell-targeting compounds or cell-specific ligands being conjugated with vesicles or carriers. Among various carriers, the CPP peptides are the potential delivery system for nucleic acids (Ignatovich et al., 2003; Järver et al., 2012; Shukla et al., 2014; Kizil et al., 2015). Forming protein-nucleic acid complex, CPPs can absorb on the surface of the double-stranded helix generally by electrostatic interaction, and such complexes can aggregate with size of up to 100 nm and have a positive charge (Rathnayake et al., 2017). CPPs were demonstrated as potential siRNA delivery systems for the suppression of gene expression. Low-cytotoxic arginine-rich CPPs were successfully used for the gene delivery to the brain (Morris and Labhasetwar, 2015).

Recombinant analog of lactaptin RL2 originates from human k-casein and demonstrates CPP-like properties (Chinak et al., 2016). Due to amphiphilic characteristics, the caseins themselves can self-assemble into micelles (Portnaya et al., 2008). In drug delivery on the organism level, casein spheres showed a superior biocompatibility being introduced orally and were successfully used as carriers of doxorubicin (Chen et al., 1987). Therefore, here we analyzed if lactaptin analog RL2 translocates nucleic acids into the cancer cells.

We observed that double stranded as well as single stranded nucleic acids were efficiently translocated into the cancer cells being in complex with RL2. The delivery of EGFP reporter gene-containing plasmid into the cells was tested for EGFP-expressing activity. Since the transcription of mRNAs in eukaryotic cells takes place strictly in the nucleus, we suggest that at least pEGFP enters the nucleus. Whether pEGFP releases from the complex in the cytoplasm and then free pEGFP is transported through the nuclear membrane or RL2-pEGFP complex enters the nucleus—this requires more investigations.

The efficiency of delivery was dependent on the RL2 amount in the complexes:RL2-rich complexes were more efficient according to the cargo functional activity. The actual process of DNA or RNA condensation with CPPs has not yet been fully understood. Our finding that pDNA : RL2 complex size is smaller than the sizes of RL2 or pEGFP alone indicates the condensation of DNA in the complex. Nowadays, the most used DNA-condensing cationic peptides in gene delivery are lysine-reach, like RPC or arginine-reach, like viral Tat peptides (Wender et al., 2000; Read et al., 2003). RL2 contains five lysines and eight arginines (**Supplementary Figure 2**), and this is likely enough to act as a DNA-condensing molecule. We suppose that the increasing of ζ-potential is specified by the rising amount of surface-exposed cationic groups of RL2 molecules in RL2:pDNA complexes.

Although stability of RL2:pDNA complexes has a few differences in the N/P range of 1.5 to 13 (**Figures 1A** and **2B**), the EGFP ectopic expression level increased with N/P increasing from 5 to 9. This could explain that RL2:pEGFP complexes with high amount of RL2 exert higher resistance to the nucleases. The same results were obtained for the siRNA delivery: despite of the findings that strong RL2:siRNA complexes were formed with N/P range from ten and higher, the efficiency of delivery increased with the increasing of RL2 in the complex. This could mean that a high RL2 concentration is needed for the successful delivery or alternatively, that complexes with high N/P value are fully shielded from extracellular enzymes or that the high RL2 concentration provides early endosome escape of siRNA also. Moreover, we suppose that RL2-mediated nucleic acids transport is non-dependent on the cargo size, but only on the RL2 amount that was confirmed by the effective delivery of big plasmid DNA and small siRNA.

We have also observed that Ca^2+^ stabilizes complexes of RL2 with nucleic acids. It is known that at low concentration divalent cations like Ca^2+^ and Mg^2+^ promote the condensation of CPP particles into agglomerates (McIntyre et al., 2017). Next, to study the plasmid protection outside the cells and intracellular release of cargo, we confirmed the pDNA release from the complexes by heparin displacement. Polyanion heparin is a suitable molecule for the modeling of nucleic acid displacement in the complexes because before the complexes reach the plasma membrane phospholipids, the delivering complexes interact with proteoglycans of the extracellular matrix, such as heparin. High heparin concentration induced total release of pDNA from the RL2:pDNA complexes.

Taken together, these findings indicate that recombinant lactaptin is able to deliver noncovalently associated nucleic acids into cancer cells *in vitro*.

## Materials and Methods

### Materials

Heparin sodium salt (H4784-1G) and CaCl_2_ were from Sigma-Aldrich. DNA Gel Loading Dye (R0611) and Lipofectamine 3000 were from ThermoFisher, molecular weight markers Sky High (10 and 500 bp) were from BioLabMix, Russia. MTT (3-(4,5-dimethyl-2-thiazolyl) -2,5-diphenyl-2H-tetrazolium bromide was from Sigma-Aldrich. Plasmid pEGFP-C1 (GenBank Accession U55763; cat 6084-1) was purchased from BD Biosciences Clontec, monoclonal anti-RL2 mouse IgG (clone F14; Biosan, Russia).

The recombinant analogue of lactaptin RL2 was obtained from *E. coli* and purified as described previously (Semenov et al., 2010). The 98% purity of the isolated protein was confirmed by RP-HPLC chromatography on C5 reverse phase column (Discovery BIO Wide Pore C5; Sigma) in water (0.5% TFA)-acetonitil solvent system using HPLC Station (Bio-RAD Laboratories) as well as by RP-HPLC on C18 (ProntosSIL) using Milichrom A-02 station (EcoNova, Russia) (**Supplementary Figure 3A**) and by SDS-PAAG electrophoresis under reduction conditions (**Supplementary Figure 3B**).

### Cell Culture

A549 human lung carcinoma cells (ATCC CCL-185) and A431 squamous carcinoma cells (ATCC CRL-1555) were grown in DMEM, supplemented with 10% fetal bovine serum (FBS), 2 mM l-glutamine, 100 U/mL penicillin, 100 mg/mL streptomycin, and 0.25 μg/mL amphotericin B, all from Gibco BRL Co. USA, at 37°C, 5% CO_2_.

### N/P Calculation

N/P values were calculated by the formula:

$$N/P=\frac{n\left(\;«+»\;\text{charged groups of }RL2\right)\times C\left(RL2\right)}{m\left(«-»\;\text{charged groups of nucleic acid}\right)\times \text{C}\left(\text{nucleic acid}\right)},\;$$

where C is the concentration (mole/L).

Amino-groups and imino-groups of amino acids in RL2 were estimated as fully protonated for lysine, arginine, and histidine under experimental conditions. Phosphate groups of nucleic acids were accounted as totally deprotonated with negative charge.

### Complexes Formation

RL2:pEGFP complexes were prepared by varying a charge ratios (N/P). RL2 (1.1 × 10^−8^ ? to 9.7 × 10^−8^ ?) and plasmid DNA pEGFP-C1 (2.9 × 10^−8^ ?) were dissolved in 25 mM MES buffer, pH 5.5, and then they were mixed and incubated for 5 min at 37°C.

For the RL2:siRNA complex formation, at first siRNA duplexes were formed. Chemically synthesized oligoribonucleotides 5′-GAACGGCAUCAAGGUGAACTT-3′ (sense) and 5′-GUUCACCUUGAUGCCGUUCTT-3′ (antisense) were from Institute of Chemical Biology and Fundamental Medicine, SB RAS, Russia. Oligoribonucleotides (1 × 10^−5^ М) were incubated in the buffer containing 50 мМ potassium acetate, 15 мМ HEPES-KOH, pH 7.4 and 1 мМ magnesium acetate for 3 min at 90°C, and then mixture was cooled to 37°C. RL2 was mixed with anti-EGFP siRNA at N/P ratios of 10 to 35 in 25 mM MES buffer, pH 5.5 for 5 min at 37°С.

For the RL2:snoRNA complex formation, artificial analogue of U25 box C/D snoRNA was synthesized as was described in the study of Stepanov et al. (2018). RL2 (0.05–0.2 mg/mL) and snoRNA (10 or 30 nM) were dissolved in 25 mM MES buffer, pH 5.5, and then were mixed and incubated for 5 min at 37°С.

### Gel Retardation Assays

Aliquots of noncovalent complexes (reaction mixture, 10 µL) were loaded onto 0.5% agarose gel (for RL2:pDNA) or onto 15% PAAG (RL2:siRNA) with DNA Gel Loading Dye. Retardation was analyzed by electrophoresis in Tris-Acetate-EDTA (TAE) buffer or Tris-Borate-EDTA (TBE) buffer. DNA was visualized with ethidium bromide by Gel Doc XR+ Image system (BioRad, USA), and siRNA was visualized with SYBR Green I Nucleic Acid Gel Stain by BioRad GelDoc XR+ Image system (BioRad, USA).

### Dynamic Light Scattering (DLS)/Determination of Particle Size, ξ-Potential, and UV-vis

The hydrodynamic diameter and zeta potential of RL2:pEGFP were determined by dynamic laser light scattering using a Zetasizer Nano ZS (Malvern Instruments Ltd, UK) at a wavelength of 623 nm and 25°C. All DLS results were calculated as the average of at least triplicate measurements and presented as mean ± SD.

UV-vis spectroscopy analysis of complexes was performed using NanoDrop 2000c (Thermo Scientific, USA).

### pDNA Delivery Microscopic Assays

A549 and A431 cells were seeded (2 x 10^4^ cells/cm^2^) on 24-well plates 24 h prior the experiment. Cell media was replaced with fresh Opti-MEM (ThermoFisher, USA) containing 2 mM l-glutamine, and pre-formed complexes were added to the cells to the final concentration of pEGFP 1 ng/µL. After 3 h, medium was replaced with complete fresh DMEM media supplemented with 10% FBS, and cells were incubated for additional 24 h. EGFP fluorescence was analyzed using fluorescent microscope (ZOE Bio-Rad, USA).

### pDNA Delivery RT-PCR Assays

After microscopic analysis, cells were washed three times with PBS and total RNA was isolated by phenol-chloroform extraction using the Lira reagent (Biolabmix Ltd, Novosibirsk, Russia) according to the manufacturer’s protocol. The quality of total RNA was assessed by agarose gel electrophoresis or capillary electrophoresis with an Agilent 2100 Bioanalyzer, using 28S/18S > 2 or RIN > 8.0 criterion. RT-PCR were performed in the one-tube reaction mixture BioMaster RT-PCR SYBR Blue (Biolabmix Ltd, Novosibirsk, Russia, www.biolabmix.ru) with gene-specific primers: EGFP 5′-GTAAACGGCCACAAGTTCAG-3′ and 5′-GGTGCGCTCCTGGACGTAGC-3′; hypoxanthine-guanine phosphoribosyltransferase (HPRT): 5′-CATCAAAGCACTGAATAGAAAT-3′ and 5′-TATCTTCCACAATCAAGACATT-3′. For RT-PCR analysis 1 µg of total RNA was used in the reaction under the following conditions: incubation at 25°C for 5 min followed by 42°C for 30 min and at 95°C for 4 min, next incubation at 59°C for 30 s, and at 72°C for 20 s followed by 30 cycles of denaturation at 95°C for 30 s, annealing at 59°C for 30 s, and elongation at 72°C for 20 s.

### Pre-Treatment With Anti-RL2 Antibodies

Cells were grown in 12-well plates up to 80% confluence before the replacement for the fresh DMEM:F12 medium supplemented with l-glutamine. Monoclonal anti-RL2 IgG (15 and 77 ng) were added to the cells and incubated for 15 min. Next, cells were treated with RL2:pEGFP complexes and incubated for 3 h. Subsequently, culture medium was replaced with fresh DMEM:F12 medium supplemented 10% FBS and l-glutamine and cells were incubated for 24 h at 37°C in CO_2_ incubator. Cells were analyzed by fluorescence microscopy (ZOE Bio-Rad, USA), and cellular lysates were used for subsequent RT-PCR analysis.

### siRNA Delivery Assay

A549 cells were seeded (2 x 10^4^ cells/cm^2^) on 12-well plates. After 24 h, cell media was replaced with fresh Opti-MEM (ThermoFisher, USA) containing 2 mM l-glutamine. Plasmid pEGFP-C1 with Lipofectamine 3000 (1 µg DNA/well) and RL2:siRNA complex were added to the cells. Cells were incubated 3 h at 37°C, and after that, FBS and antibiotics were added to the cells. Cells were incubated for 48 h and analyzed by fluorescence microscopy (ZOE Bio-Rad, USA) or by flow cytometry (BD FACSCantoII). For flow cytometry analysis cells were fixed in 5% formaldehyde for 2 h. Fluorescence was detected in the FITC channel (emission 535 nm, excitation 488 nm). Data Analysis was performed using the software package BD FACSDiva software v6.1.3.

### snoRNA Delivery Assay (MTT)

A549 cells were seeded in 96-well plates at density of 2 x 10^3^ cells per well. After 24 h cell media was replaced with 100 µL DMEM, containing 2 mM l-glutamine, and 10.5 µL RL2:U25 complexes per well. After 2 h, 50 µL DMEM supplemented with 30% FBS, 300 U/ mL penicillin, 300 mg/mL streptomycin, 0.75 μg/mL amphotericin B, and 2-mM l-glutamine was added, and cells were incubated for 48 h, and MTT analysis was performed as was described in the study of Koval et al. (2014). Cell viability was expressed as a means percentage of control ± SD for triplicate independent experiments.

### Statistical Analysis

All experiments were repeated three times independently, and data are presented as mean ± SD. The Mann-Whitney U-test was used to define statistically significant differences between groups. All error bars represent standard error of the mean. P value that was less or equal 0.05 was considered as significant.

## Data Availability

The data that support the findings of this study are available from the authors upon reasonable request.

## Author Contributions

OK designed the study and analyzed the data. OC and EG performed the experiments. IP performed determination of particle size and zeta potential. GS and EZ performed the experiments with snoRNA. The draft manuscript was prepared by OK with input from VR. All authors agreed the final version. All authors read and approved the final manuscript.

## Funding

This work funded by the Russian State funded budget project of ICBFM SB RAS AAAA-A17-117020210023-1.

## Conflict of Interest Statement

The authors declare that the research was conducted in the absence of any commercial or financial relationships that could be construed as a potential conflict of interest.

## Supplementary Material

Supplementary Figure 1 Analysis of formation of RL2:pEGFP complexes with N/P 5 – 11 by UV-vis spectroscopy.

Supplementary Figure 2 Amino acid sequence of RL2.

Supplementary Figure 3 Purity analysis of RL2. A – HPLC analysis of RL2 comparing with blanking chromatography on ProntoSIL 120-5-B – Electrophoretic analysis of RL2 in 13% SDS-PAGE in reducing conditions.
