# Supplementary figures and images for: Nucleic Acids Delivery Into the Cells Using Pro-Apoptotic Protein Lactaptin
Source: Front Pharmacol. 2019 Sep 18;10:1043. doi: 10.3389/fphar.2019.01043 (PMC6759801; doi:10.3389/fphar.2019.01043)

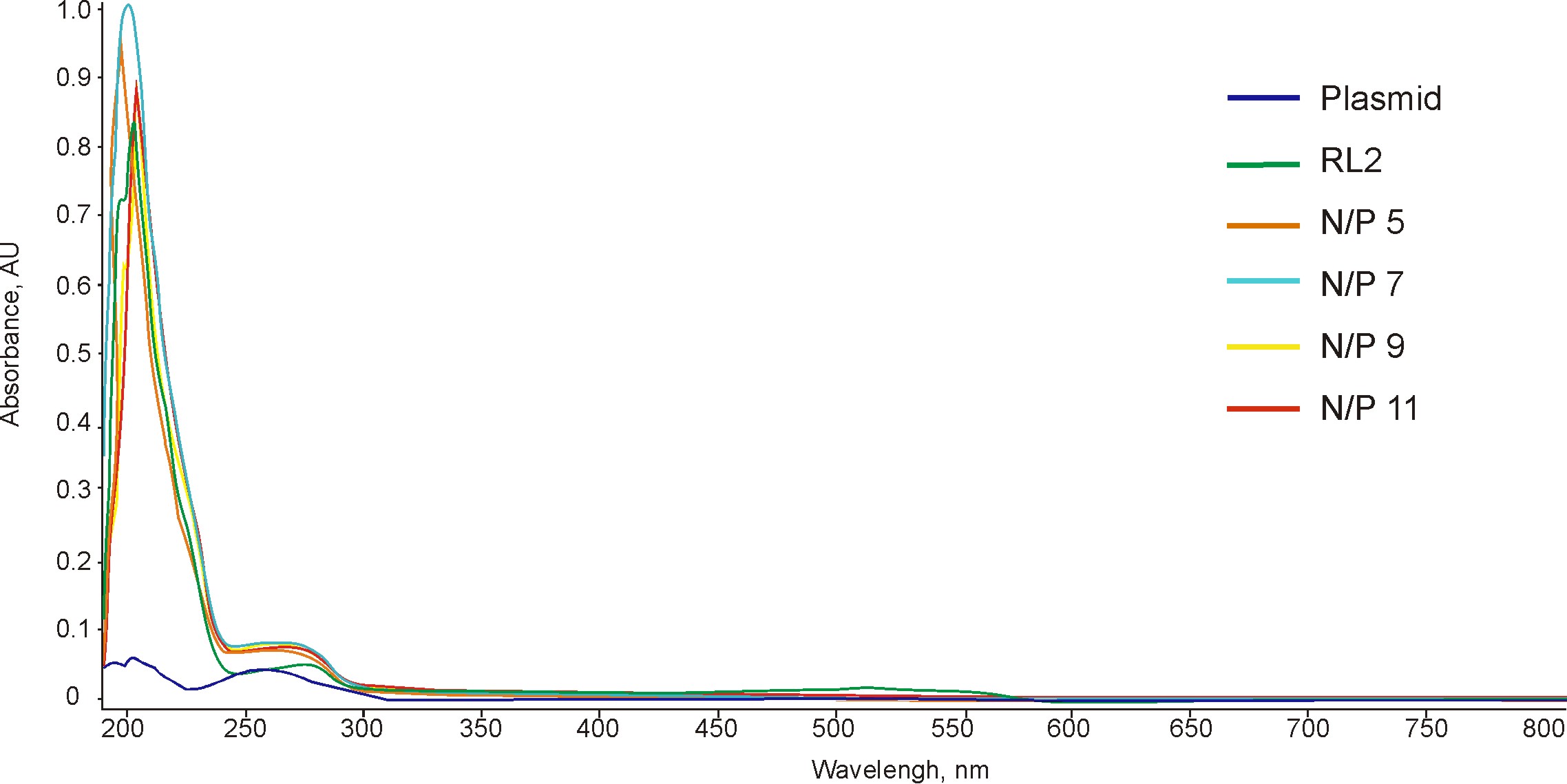

Supplement: Supplementary Figure 1 — Analysis of formation of RL2:pEGFP complexes with N/P 5 – 11 by UV-vis spectroscopy. [file Image_1.jpeg]

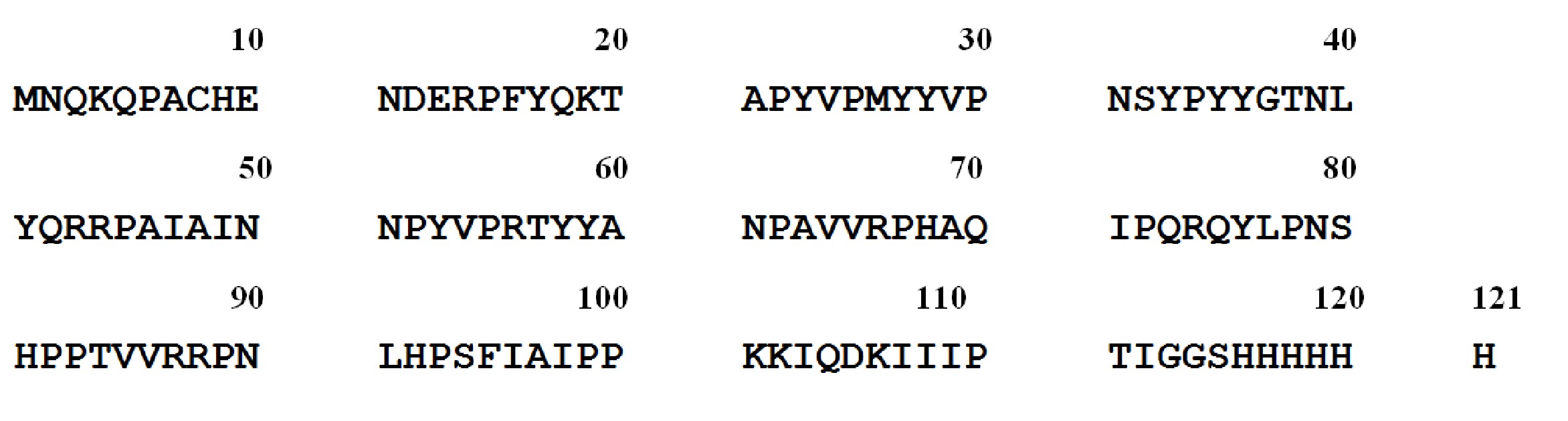

Supplement: Supplementary Figure 2 — Amino acid sequence of RL2. [file Image_2.jpeg]

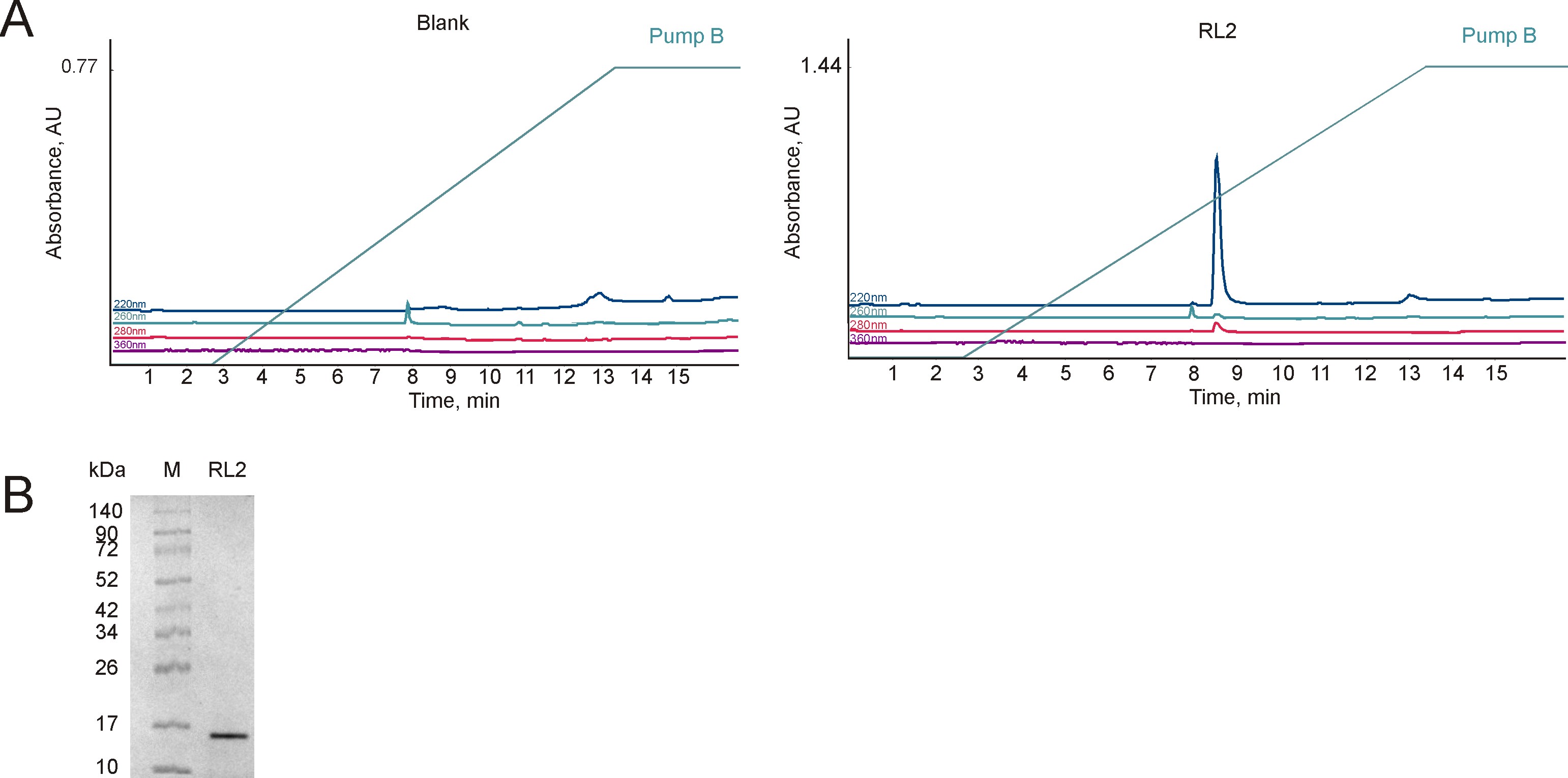

Supplement: Supplementary Figure 3 — Purity analysis of RL2. A – HPLC analysis of RL2 comparing with blanking chromatography on ProntoSIL 120-5-B – Electrophoretic analysis of RL2 in 13% SDS-PAGE in reducing conditions. [file Image_3.jpeg]
